# A review of ablative techniques in the treatment of breast fibroadenomata

**DOI:** 10.1186/s40349-016-0045-z

**Published:** 2016-01-19

**Authors:** Mirjam C. L. Peek, Muneer Ahmed, Sarah E. Pinder, Michael Douek

**Affiliations:** Division of Cancer Studies, King’s College London, Guy’s Hospital Campus, Great Maze Pond, London, SE1 9RT Great Britain

**Keywords:** Fibroadenomata, High-intensity focused ultrasound, Laser ablation, Cryo-ablation, Ablative techniques

## Abstract

**Introduction:**

Breast fibroadenomata (FAD) are benign lesions which occur in about 10 % of all women. Diagnosis is made by triple assessment (physical examination, imaging and/or histopathology/cytology). For a definitive diagnosis of FAD, the treatment is conservative unless the patient is symptomatic. For symptomatic patients, the lumps can be surgically excised or removed interventionally by vacuum-assisted mammotomy (VAM). Ablative techniques like high-intensity focused ultrasound (HIFU), cryo-ablation and laser ablation have also been used for the treatment of FAD, providing a minimally invasive treatment without scarring or poor cosmesis. This review summarises current trials using minimally invasive ablative techniques in the treatment of breast FAD.

**Methods:**

A comprehensive review of studies using minimally invasive ablative techniques was performed.

**Results:**

There are currently several trials completed or recruiting patients using HIFU, cryo-ablation and laser ablation in the treatment of breast FAD. The results look very promising but cannot be compared at this point due to heterogeneity between studies.

**Conclusion:**

Minimally invasive ablative techniques like HIFU, cryo-ablation and laser ablation are promising in the treatment of breast FAD. Future trials should be randomised and contain larger numbers of patients to determine the effectiveness of ablative techniques with more precision.

## Background

Breast fibroadenomata (FAD) are solitary, smooth, mobile benign breast lesions that can occur at any age but most often during the second and third decades [1–3]. Regression or complete resolution is seen in up to 59 % of cases within 5 years [1]. FAD consist of combined proliferation and epithelial and fibroblastic tissue elements which are oestrogen-dependent and slowly growing [3]. FAD are believed to arise from breast lobulesin the ductal system of the breast [2] FAD are usually diagnosed by standard triple assessment which entails a physical examination, imaging by ultrasound and/or mammogram and if the patient is 25 years or older, a cytological or histological conformation of the diagnosis [2, 3]. In the UK, management of FAD is achieved via a multidisciplinary approach to ensure a more consistent approach to recommendation for surgical and interventional excision [4]. Management of non-symptomatic FAD is conservative once definitive diagnosis is made [2]. Management of symptomatic FAD consists of either conservative treatment, surgical excision or vacuum-assisted mammotomy (VAM) [2]. These options are discussed with the patient, bearing in mind the severity of symptoms against the scarring or discomfort from treatment of a benign condition [2]. In patients with large FAD or fast growing lesions, excision should be recommended [2, 3]. Minimally invasive ablative techniques offer the opportunity to treat FAD without scarring and the ability to image the progress during treatment. We reviewed the current evidence of non- or minimally invasive ablative techniques in the treatment of breast FAD.

### High-intensity focused ultrasound

**Fig. 1:**
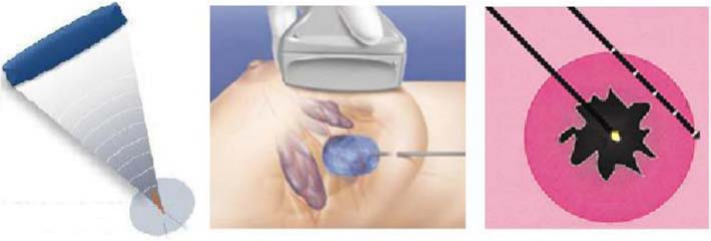
Treatment overviews: (*left*) high-intensity focused ultrasound, (*middle*) cryo-ablation and (*right*) laser ablation [23, 33, 34]

High-intensity focused ultrasound (HIFU) is a completely non-invasive ablative technique (Fig. 1) [5]. During HIFU, an ultrasound (US) beam generated by a piezoelectric US transducer propagates through tissue as a high-energy pressure wave [6]. The beam is focused onto the targeted tissue, and the energy from the beam elevates the temperature up to 60–95 °C within a few seconds without causing damage to direct adjacent tissues, allowing a focused ablation leading to protein denaturation and coagulative necrosis [6, 7]. Seven studies have been performed of which one is currently recruiting patients for a HIFU trial; the protocols and/or results are summarised in Table 1.

**Table 1:** High-intensity focused ultrasound (HIFU) ablation studies treating fibroadenomata

Study	Number	Size (cm)	Age (years)	Device	Follow-up (months)	Therapeutic efficacy	Complications	Time (min)
Hynynen (2001)	9 (11)	1.9 ± 1.5 cm^3^ (0.7–6.5)^a^	29 (19–38)^b^	InSightec (transducer diameter 100 mm and radius curvature 80 mm)	6 (18–48)	Complete necrosis (*n* = 6), partial necrosis (*n* = 2), minor necrosis (*n* = 1) and no response (*n* = 2)	Oedema, tenderness	–
Tempany (2005)	102	–	–	InSightec (−)	–	–	–	–
Kovatcheva & Boulanger (2014)	42 (51)	3.9 ml (0.3–19.7)^a^	32 (16–52)^a^	EchoPulse (transducer diameter 60 mm, 3 MHz)	12	Reduction of 33.2 ± 19.1 % at 2 months, 59.2 ± 18.2 % at 6 months and 72.5 ± 16.7 % at 12 months	Skin burn (*n* = 3), hyperpigmentation (*n* = 1)	118 (60–255)^a^
Cavallo-Marincola (2014)	10	–	26 (18–34)^a^	JC HIFU (−)	–	50 % reduction after in maximum diameter after 3 months	Swelling, hardness of area	57.2 (40–100)^a^
Hahn (2016)	27	–	–	EchoPulse (transducer diameter 60 mm, 3 MHz)	12	–	–	–
Douek (2016)	50	–	–	EchoPulse (transducer diameter 60 mm, 3 MHz)	12		–	–
Brenin (2016)	20	–	–	EchoPulse (transducer diameter 60 mm, 3 MHz)	12		–	–

^a^Mean ± SD (range)

^b^Median (range)

Hynynen et al. [8] evaluated the feasibility of HIFU using magnetic resonance imaging (MRI) guided HIFU system (GE Medical Systems, Milwaukee, USA) for the treatment of 11 FAD in nine patients (median 29, range 19–38 years) with a mean volume of 1.9 ± 1.5 cm^3^ (range 0.7–6.5 cm^3^). Complete response was defined as a decrease in volume of >90 %, partial response as a decrease between 50–90 % and minor response as a decrease between 10 and 49 %. T1-weighted MRI showed complete (*n* = 6), partial (*n* = 2), minor (*n* = 1) and no response (*n* = 2) to HIFU treatment, and T2-weighted MRI showed a mean volume decrease to 1.3 ± 1.1 cm^3^ at 6 months post-treatment. On thermal imaging, hotspots of 73.6 % (743/1010) of all sonications were visible and a temperature elevation between 12.8 and 49.9 °C were seen in MRR imaging. Pain was recorded as slight (*n* = 4), moderate (*n* = 2) or severe (*n* = 1). Tenderness was common up to 10 days and oedema up to 2 days post-treatment.

Tempany et al. [9] evaluated the safety and efficacy of HIFU treatment in 102 patients using the MRI-guided InSightec device (GE Medical Systems, Milwaukee, USA). The goal was to obtain a decrease in size of ≥50 % on physical examination and ≥65 % on MRI. Inclusion criteria were patients of ≥18 years with a histologically confirmed single FAD, visible on non-contrast MRI with a size of ≥0.5 cm. Follow-up period was not documented.

Kovatcheva and Boulanger et al. [10] included 42 women with 51 FAD in an uncontrolled open-label prospective multicentre study using the Echopulse device (Theraclion Ltd., Malakoff, France). Eight patients (19 %) received a second treatment because symptoms persisted and/or volume reduction after 3 months post-treatment was ≤50 %. Patients’ mean age was 32 years (range 16–52 years), and average treatment time was 118 min (range 60–255 min). Mean FAD volume on US was 3.89 ml (range 0.34–19.66 ml), and a volume reduction of 33.2 % (SD 19.1 %) was visible at 2 months, 59.2 % (SD 18.2 %, *p* < 0.001) at 6 months and 72.5 % (SD 16.7 %, *p* < 0.001) at 12 months. Mean visual analogue pain scale at the end of treatment was 29.7 ± 27.5 mm (range 0–80 mm). Post-treatment complications were superficial skin burns (*n* = 3), hyperpigmentation (*n* = 1) and a palpable subcutaneous induration between the FAD and the skin (*n* = 1).

Cavallo Marincola et al. [11] presented the results of using the model JC HIFU device in ten patients with breast FAD. Patients’ mean age was 26 years (range 18–34 years), and the treatment time between the first and last sonication was 57.2 min (range 40–100 min). At 3 months follow-up, a 50 % reduction in maximum diameter was seen on US. No adverse events were reported apart from mild swelling and hardness of the treated area.

In the mono-centre, open-label uncontrolled study by Hahn et al. [12], 27 patients were recruited to evaluate the efficacy of HIFU using the Echopulse device (Theraclion Ltd., Malakoff, France). The secondary outcome measure was the tolerability of HIFU treatment. Patients were included if ≥18 years with at least one diagnosed FAD based on triple assessment and a maximum diameter of 2.5 cm. Patients were followed-up for 12 months.

Douek et al. [13, 14] performed a feasibility study of 50 patients to assess the treatment of FAD with a circumferential HIFU treatment using the Echopulse device (Theraclion Ltd., Malakoff, France). The primary outcome measure was the change in volume of FAD as recorded on US, and secondary outcome measures include complications, patient-recorded outcome measures, mean treatment time and cost-effectiveness of HIFU. Patients were eligible if ≥18 years with FAD diagnosed according to local hospital protocol, visible on US, and for patients of ≥25 years, cytological or histological confirmation was required. Patients were followed-up at 2 weeks, 3, 6 and 12 months.

Benin et al. [15] designed a FDA approved feasibility study with 20 patients to determine the safety and efficacy of the Echopulse device (Theraclion Ltd., Malakoff, France). Primary outcome measures were the change in volume of the FAD measured by US, size of FAD on physical examination, patient-recorded outcomes by measuring patients rated pain and response to satisfaction questionnaires. The secondary outcome measure was the incidence of adverse events. Patients were eligible if ≥18 years, with a palpable histological confirmed FAD with a size ≥1 cm and volume between 2 and 10 cm^3^. Patients are followed-up at 3, 6 and 12 months.

In general, studies so far included small patient numbers and none were controlled. Further patient follow-up is required on existing studies. Post-treatment complications were mild, and treatment times lasted between 1 and 2 h.

### Cryo-ablation

Cryo-ablation uses freezing instead of heating in the treatment of breast lesions (Fig. 1). Modern cryo-ablation has been used for more than 20 years. It is accomplished by inserting a cryo-probe under US guidance into the target tissue. Liquid nitrogen or Argos gas is then inserted into the cryo-probe allowing it to flow towards the target tissue. The freezing process involves two phases: freezing and thawing. Four mechanisms destroy the tumour cells: direct damage by (1) intracellular ice crystal formation resulting in disruption of cell membranes and (2) osmotic dehydration and indirect damage by coagulation of blood resulting into (3) ischemia and (4) immunologic response. The treatment has good precision and control because formation of the ice ball can be clearly visualised using US [16, 17]. Seven studies performed trials or have published results of trials using cryo-ablation in the treatment of FAD. These studies are summarised in Table 2.

**Table 2:** Cryo-ablation studies treating fibroadenomata

Study	Number	Size (cm)^a^	Age (years)^a^	Device type	Therapeutic efficacy	Complications	Follow-up (months)^a^	Time (min)^a^
Caleffi (2004)	102 (124)	1.4 ± 0.6 vs 2.1 ± 0.8	38 (45 vs 34 ± 13)	Visica, 2.4 mm cryo-needle, −160 °C	Double HI-Freeze 67 % still palpable after 12 months, Tailored freeze volume reduction 91 % after 12 months	Tape blisters (*n* = 6), keloid (*n* = 2), ecchymosis, discomfort, oedema	12	16.1 vs 14.7 ± 3.3
Littrup (2005)	29 (42)	4.2 ± 4.7	26.6 (13–50)	Visica, 2.4 mm cryo-needle, −187 °C	Reduction in tumour volume 73 % after 12 months	Hypo-pigmentation (*n* = 3)	12	–
Kaufman (2004)	57 (70)	2.1 (0.7–4.2)	35 ± 13 (13–66)	Visica, 2.4 mm cryo-needle, −186 °C	Reduction in tumour volume 89 % after 12 months	Moderate-severe pain (*n* = 2), hematoma (*n* = 2), skin injury (*n* = 4), tape blisters (*n* = 7), pigmentation (*n* = 3), keloid (*n* = 2), nipple retraction (*n* = 1)	12	14.8 ± 3.3
Kaufman (2005)	29 (32)	2.1 ± 0.8 (0.8–4.2)	35 (13–66)	Visica, 2.4 mm cryo-needle, −186 °C	Median reduction in tumour volume 89 % at 12 months and 99 % at most recent follow-up	Tenderness (*n* = 2), pain (*n* = 1)	31.2 (15.6–45.6)	14.3 (6–20.1)
Nurko & Edwards (2005)	444	1.8	–	Visica, 2.7 mm cryo-probe, −160 °C	Mean reduction in tumour volume 51 % after 6 months and 97 % after 12 months.	–	12	–
Golatta & Klein (2014)	60	1.2 ± 0.9 cm^3^	–	IceSense3, up to 3.5 mm cryo-probe, −196 °C	Completely resolved FAD 93 % after 12 months	Induration of breast (*n* = 1)	12	–

^a^Mean ± SD (range)

Klein et al. [18] performed a study with 54 patients to determine whether the ice-sense cryo-ablation system (IceCure Medical Ltd., Tel Aviv, Israel) was safe and effective in the treatment of benign breast lesions like FAD. The outcome measures were engulfment of the iceball, as seen on US, and adverse events. Patients were eligible if ≥18 years, with histologically proven FAD, visible on US and a size between 0.5 and 3.0 cm. Follow-up was performed for 12 months.

Golatta et al. [19] performed the multicentre open-labelled non-randomised CRYSTAL study using the IceSense3 (IceCure Medical, Caesarea, Israel) in the treatment of FAD. Data was collected from 60 procedures using a freeze-thaw-freeze technique, with 58 cases completing a 12-month follow-up. Engulfment of the lump was achieved in 98.3 % with an average first freezing cycle of 1.44 min and second cycle of 1.30 min. One mild and resolved adverse event (induration of the patient’s breast) occurred. Prior to treatment, 76 % (46/60 cases) of FAD were palpable. Twenty-eight percent (17/60 cases) reported mild to moderate pain prior to treatment. At 1 year, 22 % (13/58 cases) were palpable and 2 % (1/58 cases) reported mild to moderate pain. Cosmetic results were described as good–excellent in 100 % by physicians and 97 % by patients. Mean FAD volume was 1.2 cm^3^ (SD 0.9 cm^3^) prior to treatment, and FAD were completely resolved in 93 % at 1 year post-treatment.

Kaufman et al. performed a prospective non-randomised trial using cryo-ablation in the treatment of benign breast lesions. One article [20] described all included patient results, and three articles [21–23] described the results of patients with FAD. The mean age of 57 patients with 70 FAD who were included (mean ± SD (range)) was 35 ± 13 years (13–66 years). Patients were eligible if evaluated by physical examination, mammography, ultrasound and histology confirming the diagnosis. The Visica device (Sanarus Medical, Pleasanton, California) was used with a freeze-thaw-freeze technique. The mean treated lesion diameter as seen on US was 2.1 ± 0.08 cm (range 0.7–4.2 cm). At 12 months, 11 % of median tumour volume was remaining and 75 % of tumours became non-palpable. A good or excellent cosmesis was achieved in 91 % at 12 months and unsatisfactory in 9 %. Post-operative complications were local swelling (all), ecchymosis (all), moderate to severe pain (*n* = 2), mild hematoma (*n* = 32), mild skin injury (*n* = 4), minor tape blisters (*n* = 7), minor pigmentation of the skin (*n* = 3), keloid at puncture side (*n* = 2) and transient nipple retraction (*n* = 1). Long-term follow-up [22] (2.6 years (range 1.3–3.8 years)) in 29 patients (32 FAD) showed palpability decrease from 84 % initially to 16 % at the most recent follow-up. On US, the median reduction in volume of the residual FAD debris was 89 % at 12 months and 99 % at the most recent follow-up. Two types of complications were reported (*n* = 2) and cyclic focal pain (*n* = 1). Overall satisfaction (scales 1–5) by physicians was on average 4.8 and 4.6 by patients. Cosmesis was 100 % by both patients and physicians.

A multicentre trial by Edwards et al. [24] used the Visica cryo-ablation system (Sanarus Medical, Pleasanton, California). Two freeze-thaw cycles were used, and follow-up was performed at 6 and 12 months. At 53 sites, 310 FAD were treated with a mean diameter of 1.8 cm. FAD were palpable at pre-treatment in 77 %, post-treatment at 3 months 50 % and at 6 months in 33 % of patients. Average residual lesion volume was 49 % at 6 and 3 % at 12 months. Complications were infection (2 %), ecchymosis (unknown), hematoma (amount comparable to surgical excision), tape blisters (5 %) and depigmentation (1 %). Ninety-two percent of patients were satisfied with the procedure and 91 % would recommend treatment.

Littrup et al. [25] performed a study with 29 patients and 42 FAD to assess imaging and clinical outcomes of cryo-ablation for breast FAD. The US guided Visica treatment system (Sanarus Medical, Pleasanton, California) was used with a freeze-thaw-freeze cycle. Mean patient age was 26.6 years (range 13–50 years) with an average volume of 4.2 ± 4.7 cm. At 6 months, four FAD showed ‘fragmentation’ and at 12 months, five FAD could no longer be identified. A reduction of 73 % to 0.7 ± 0.8 cm (*P* < 0.001) was found. All patients were happy with the cosmesis, and in three patients, scarring at the insertion site leads to hypo-pigmentation which resolved between 6 and 12 months. One patient developed a keloid and at the end of the trial, two patients underwent surgical excision.

Caleffi et al. [26] used the Visica system (Sanarus Medical, Pleasanton, California) to carry out interstitial US-guided cryo-ablation of 124 benign breast lesions in 102 patients. The Double HI-Freeze technique was used for 42 breast lesions (unknown amount of FAD) with mean age 45 years and mean size 1.4 ± 0.6 cm; patients were treated within an average 16.1 min. The Freeze technique was used in 82 breast lesions (70 FAD, five fibrocystic changes and seven other benign changes) with mean age 34 ± 13 years, mean size 2.1 ± 0.8 cm and a mean time of 14.7 ± 3.3 min. Within the Double HI-Freeze group, 14 lumps were palpable pre-treatment and at 1 year post-treatment, 67 % (24/36 lesions) were palpable. Within the Tailored freeze group, a volume reduction of 91 % at 12 months was visible. Reported complication were ecchymosis, discomfort, oedema, tape blisters (*n* = 6) and keloid (*n* = 2). Patient satisfaction was excellent in 92 % of patients.

Nurko et al. [27] developed the multicentre FibroAdenoma Cryoablation Treatment (FACT) trial to evaluate the cryo-ablation technique. The Visica treatment system (Sanarus Medical, Pleasanton, California) was used with a freeze-thaw-freeze technique. Four hundred forty-four FAD were treated with a mean diameter of 1.8 cm. Prior to cryo-ablation, 75 % of FAD were palpable and 100 % were visible on US. At 6 months, 46 % of cases (110/237) were palpable and 36 % (79/219) visible on US and at 12 months, 35 % of cases (29/82) were palpable and 29 % visible on US (21/71). Mean treatment time was 31 min with a mean freezing time of 22 min. At 6 and 12 months, acute complications (no details reported) had resolved without consequences apart from residual pain in 15 % (37/242 cases) at 6 months, and 25 % (22/87 cases) at 12 months. Patient satisfaction was 91 % (216/235 cases) at 6 months and 88 % (74/84 cases) at 12 months.

In general, the cryo-ablation studies contained larger numbers compared to HIFU. Post-treatment complications were more serious but less frequent compared to HIFU and treatment times were about 15 min.

### Laser ablation

In laser ablation (Fig. 1), lesions are destroyed using direct heating with low-power laser light energy delivered via thin optical fibres which are percutaneously inserted under US or MRI guidance [28]. Upon absorption in the tissue, heat is produced, inducing lethal thermal injury by changing the optical characteristics of the tissue [29]. The level of necrosis is dependent on the temperature and the ablation time. Laser ablation can induce very precise necrosis. The size and shape of thermal lesions are difficult to predict, however owing to biologic variability, fibre tip charring and changing optical and thermal properties of the tissue during interstitial laser photocoagulation [28]. Three studies have been performed, of which one is currently collecting data, for trials using laser ablation in the treatment of FAD (Table 3).

**Table 3:** Laser ablation studies treating fibroadenomata

Study	Number	Size (cm)^a^	Age (years)^a^	Type	Therapeutic efficacy	Complications	Follow-up (months)^a^	Time (min)^a^
Basu (1999)	27	<2	21.8 (14–35)	Nd:YAG (1064 nm, 2 W) diameter 600 μ	60–70 % decrease histologically and 40–50 % decrease on ultrasound	Epithelial skin breakdown and hyperpigmentation (*n* = 8)	2	5
Lai (1999)	24 (29)	2.5 (1.4–3.5)	26 (18–42)	Diomed 25 (805 nm, 2.5 W)	Mean reduction in size 38 % after 3 months, 60 % after 6 months and 100 % after 12 months	Discomfort, swelling and tenderness (*n* = 20), bruising (*n* = 4), skin burn (*n* = 3), oily discharge (*n* = 1)	12	–
DeLay (2008)	500	–	–	Novilase	–	–	–	–

^a^Mean (range)

DeLay et al. [30] performed an observational study to monitor long-term safety and effectiveness of the Novilase device (Novian Health Ltd., Chicago, USA) in 500 patients. Patients were included if ≥18 years with core needle biopsy confirmed FAD, detected either by physical examination or imaging, ≤2 cm in diameter and located ≥0.5 cm away from the skin and chest wall. No follow-up period was reported.

Basu et al. [31] included 27 patients in an uncontrolled prospective study to evaluate the effects of interstitial laser hyperthermia in FAD of the breast. For the procedure, the Lasermatic (model 5050–23, Combolaser, Helsinki, Finland) was used with ND:YAG bare quartz fibres of 600 μ in diameter. Mean age was 21.8 years (14–35 years). All patients experienced a warm sensation locally during the procedure, and immediately post-treatment US showed a hyper-echoic zone with a narrow rim of hypo-echogenicity (0.3–0.5 cm). Blanching of the skin at the needle insertion site was seen in eight patients, followed by skin breakdown and hyperpigmentation during follow-up. At 2 weeks, all lumps were tender and less mobile and US showed a decrease in size and a narrower hypo-echoic rim. At 4 weeks, US showed a heterogeneous echo pattern and at 8 weeks, further reduction of the lump was seen. Histopathology in ten patients showed fibrotic tissue, and a decrease in size was found clinically (60–70 % reduction, mean 2.6 ± 0.8 cm to 1.3 ± 0.6 cm) and on US (40 50 % reduction, mean 2.2 ± 1.0 cm to 0.7 ± 0.4 cm).

Lai et al. [32] evaluated the feasibility of laser ablation using the semiconductor diode laser (Diomed 25, Diomed Ltd., Cambridge, UK) as a minimal invasive technique for FAD. Twenty-four patients with 29 FAD were treated with a median age of 26 years (18–42 years) and medium size of 2.5 cm (1.4–3.5 cm). Size reduction was seen in 28 lesions, and six patients had surgical excision post-treatment. Median reduction in size was 38 % at 3 months, 60 % at 6 months and 100 % at 12 months. Lumps were no longer palpable and no FAD increased in size. In 11/17 lesions which showed strong enhancement on pre-MRI, zones of non-enhancement post-treatment were visible. Reported complications were discomfort, local swelling and tenderness (*n* = 20), bruising (*n* = 4), small skin burn around needle insertion point (*n* = 3) and a persistent oily discharge for 3 weeks (*n* = 1).

In general, data on laser ablation therapy is available from only a few studies although these included reasonable numbers of patients. Post-treatment complications were frequent and severe compared to HIFU and cryo-ablation. Treatment times were quick, approximately 5 min.

## Discussion

The use of minimally invasive techniques like laser, cryo- and HIFU ablation enables the patient to undergo treatment without surgery and general anaesthesia and the risk of complications accompanied with the currently used techniques. Further advantages are the possibility of defining the target intraoperatively, an absence of scarring and therefore an improved cosmesis and reduced recovery time which could result in economic benefits. The disadvantage is that the FAD is not removed but will gradually decrease in size in the months following treatment. Patients must therefore be well informed to prevent anxiety regarding the lump. Furthermore, histological confirmation cannot be obtained during treatment as no specimen is removed. Histological diagnosis and a treatment plan should therefore be obtained before ablative treatment. If no histology is obtained in advance, a risk exists that the lesion might actually be a malignant lesion or other benign condition.

Objectively, only the cryo-ablation and laser ablation techniques can be compared since for HIFU only three out of seven studies have currently published results, and one trial has presented preliminary results. For cryo-ablation, six of seven studies, and in laser ablation, two of three studies, have published results. Large trials are required to objectively compare these minimally invasive techniques.

Looking at the efficacy of the treatment, in HIFU [8], a decrease in volume of 32 % was seen after 6 months. In cryo-ablation [20, 24–26], there was a mean decrease in volume of 40.6 % at 6 months and 87.3 % at 12 months. For laser ablation [32], the mean decrease in size was 60 % at 6 months and 100 % at 12 months. Therefore, laser ablation shows the best potential. However, due to the limited amount of results and the inhomogeneity of the trials, no conclusions can be made. The lower volume reduction seen with HIFU can be explained by the absence of a needle or probe inserted into the FAD which directly heats or cools the surrounding tissue. With HIFU, due to this absence, there is greater care needed to avoid injury to surrounding tissues or the skin.

Complications such as oedema, pain, tenderness and bruising are common in all techniques; tape blisters, hamartoma and keloids occur in cryo-ablation; and the tape blisters result in depigmentation of the skin near the needle insertion point. In laser ablation, skin burns were more common; however, these could occur in HIFU as well.

In general, no distinct difference can be identified between the three techniques; efficacy and complications are similar in all techniques from these preliminary series. More results, from large trials, are needed to give more conclusive outcomes.

## Conclusions

Minimally invasive ablative techniques like HIFU, cryo-ablation and laser ablation are promising in the treatment of breast FAD. Future trials should be randomised and contain larger numbers of patients to determine the effectiveness of ablative techniques with more precision.

## Abbreviations

FAD: fibroadenoma
HIFU: high-intensity focused ultrasound
MRI: magnetic resonance imaging
US: ultrasound
VAM: vacuum-assisted mammotomy
